# Identification of Anomalies in Lung and Colon Cancer Using Computer Vision-Based Swin Transformer with Ensemble Model on Histopathological Images

**DOI:** 10.3390/bioengineering11100978

**Published:** 2024-09-28

**Authors:** Abdulkream A. Alsulami, Aishah Albarakati, Abdullah AL-Malaise AL-Ghamdi, Mahmoud Ragab

**Affiliations:** 1 Department of Information Technology, Faculty of Computing and Information Technology at Alkamil, University of Jeddah, Jeddah, Saudi Arabia; aaalsulami6@uj.edu.sa; 2 Department of Mathematics, Faculty of Science, University of Jeddah, Jeddah, Saudi Arabia; aaalbarakati1@uj.edu.sa; 3 Information Systems Department, Faculty of Computing and Information Technology, King Abdulaziz University, Jeddah 21589, Saudi Arabia; aalmalaise@kau.edu.sa; 4 Information Systems Department, School of Engineering, Computing and Design, Dar Al-Hekma University, Jeddah 22246, Saudi Arabia; 5 Information Technology Department, Faculty of Computing and Information Technology, King Abdulaziz University, Jeddah 21589, Saudi Arabia; 6 Mathematics Department, Faculty of Science, Al-Azhar University, Naser City, Cairo 11884, Egypt

**Keywords:** lung and colon cancer, ensemble learning, swin transformer, histopathological images, walrus optimization algorithm

## Abstract

Lung and colon cancer (LCC) is a dominant life-threatening disease that needs timely attention and precise diagnosis for efficient treatment. The conventional diagnostic techniques for LCC regularly encounter constraints in terms of efficiency and accuracy, thus causing challenges in primary recognition and treatment. Early diagnosis of the disease can immensely reduce the probability of death. In medical practice, the histopathological study of the tissue samples generally uses a classical model. Still, the automated devices that exploit artificial intelligence (AI) techniques produce efficient results in disease diagnosis. In histopathology, both machine learning (ML) and deep learning (DL) approaches can be deployed owing to their latent ability in analyzing and predicting physically accurate molecular phenotypes and microsatellite uncertainty. In this background, this study presents a novel technique called Lung and Colon Cancer using a Swin Transformer with an Ensemble Model on the Histopathological Images (LCCST-EMHI). The proposed LCCST-EMHI method focuses on designing a DL model for the diagnosis and classification of the LCC using histopathological images (HI). In order to achieve this, the LCCST-EMHI model utilizes the bilateral filtering (BF) technique to get rid of the noise. Further, the Swin Transformer (ST) model is also employed for the purpose of feature extraction. For the LCC detection and classification process, an ensemble deep learning classifier is used with three techniques: bidirectional long short-term memory with multi-head attention (BiLSTM-MHA), Double Deep Q-Network (DDQN), and sparse stacked autoencoder (SSAE). Eventually, the hyperparameter selection of the three DL models can be implemented utilizing the walrus optimization algorithm (WaOA) method. In order to illustrate the promising performance of the LCCST-EMHI approach, an extensive range of simulation analyses was conducted on a benchmark dataset. The experimentation results demonstrated the promising performance of the LCCST-EMHI approach over other recent methods.

## 1. Introduction

According to the World Health Organization (WHO), cancer causes the most number of deaths across the globe; it is estimated that a total of 28.4 million cases will be diagnosed with cancer globally by 2040 [1]. Excessive weight gain, heavy alcohol usage, a sedentary lifestyle, and an unhealthy diet are some other risk factors for cancer. Cancer cannot be characterized as a solitary disorder, but a combination of a broad variety of diseases. Amongst its different types, colon and lung cancer is a life-threatening cancer and is responsible for about 10% of cancer-associated deaths internationally [2]. Mildly invasive technologies, like histopathology, are necessary for precise recognition of these diseases and improved treatment quality. However, it is challenging to achieve the precise classification of these tumours using non-invasive techniques [3]. Their initial identification, from a pool of different types of cancers, plays a crucial role in combating the cancer. Conventional medical imaging technologies have been used for a long time to detect colon and lung cancer. CT, MRI, and PET remain the primary clinical techniques applied in the diagnosis of this cancer type [4]. Obtaining high-resolution images is a critical criterion for detecting cancer at its initial phase and considerably improving the efficacy of the treatment process. Histopathological imaging is a technology for accurate detection of all types of cancers since it offers an in-depth and detailed analysis of the disease [5]. These are the most significant sources of essential information in the medical field that help pathologists identify cancer [6].

Histopathological images are typically employed in cancer classification procedures as they provide more complete information for identification than recent medical imaging technologies. Pathologists might select appropriate treatment plans for the type of cancer, depending on these histopathological images in combination with genomic information [7]. With the deployment of digital sliding scanners and their application in various medical departments, the digitization of histopathological slides such as whole-slide images (WSIs) into gigapixel images is becoming highly predominant. The digitization process of the histopathological images, called digital pathology, forms a unique method for collecting image data for artificial intelligence (AI) and deep learning (DL) methods [8]. This is mainly because of the growth in DL techniques, particularly convolutional neural networks (CNNs), which have attained exceptional outcomes in several computer vision (CV) tasks. In recent times, the involvement of the Computer-Aided Diagnosis (CAD) process in colon and lung cancer recognition has achieved major success and better precision than humans in a quicker time [9]. The AI technique presents solutions to address the limitations of conventional methods using technologies like fuzzy methods, SVM, expert systems, ANNs, and GA for disease diagnosis using clinical images [10]. Moreover, there is a need for the development of new techniques for identifying and classifying the LCC histopathological images for improved identification accuracy and patient outcomes.

This article presents a novel technique called Lung and Colon Cancer using a Swin Transformer with an Ensemble Model on Histopathological Images (LCCST-EMHI). The proposed LCCST-EMHI method is used to design the DL model for the detection and classification of the LCC using histopathological images. In order to accomplish this, the LCCST-EMHI method utilizes the bilateral filtering (BF) technique to get rid of the noise. In addition to this, the Swin Transformer (ST) approach is deployed as the feature extraction method. For the LCC recognition and identification process, an ensemble deep learning classifier is employed using three techniques: bidirectional long short-term memory with multi-head attention (BiLSTM-MHA), Double Deep Q-Network (DDQN), and sparse stacked autoencoder (SSAE). Eventually, the hyperparameter selection of the three DL models is implemented utilizing the walrus optimization algorithm (WaOA) method. In order to illustrate the promising performance of the LCCST-EMHI approach, an extensive range of simulation analyses were conducted using a benchmark dataset.

## 2. Review of the Literature

In the method presented by Chhillar and Singh [11], after the completion of the pre-processing phase, the proposed model retained both colour and texture features using the Color and Haralick histogram feature extraction methods correspondingly. The attained features were connected to create a solitary feature set. In this study, three feature groups (colour, texture, and united features) were approved for the LightGBM classifiers for the purpose of classification. Muniasamy et al. [12] designed the pre-trained EfficientNetB7 method to identify the primary cancer types in LC histopathology images so as to provide initial treatment for LC patients. The goal of the study was to determine the presented model’s performance using precision measures. For this study, a dataset containing 15,000 LC histopathology images was used. EfficientNetB7, a superior form of CNN, was pre-trained with ImageNet for TL and trained using this dataset. A precision metric was utilized in this study for analyzing the presented method. Singh and Singh [13] presented an ensemble classification using three different methods such as the LR, SVM, and RF methods. The predictions from every classification method were combined with the help of the usual voting process to form an ensemble classification. The deep features from lung and colon images were removed using two techniques i.e., VGG16 and LBP. With the incorporation of VGG16 and LBP features, significant features were produced at early stages for ensemble classification. Tummala et al. [14] recently stated that the DL technique exhibits optimistic outcomes in the medical field. This characteristic facilitates initial treatment and diagnosis based on disease severity. After testing the EffcientNetV2 methods five times with cross-validation, the author presented an automatic technique for the identification of the lung and colon tumour subtypes from LC25000 HI. In this study, an advanced DL structure was used based on the progressive learning and compound scaling principles while EfficientNetV2 small, large, and medium methods were used for comparison.

Iqbal et al. [15] presented a novel technique named ColonNet, a heteromorphous CNN with a feature embedding approach, designed for evaluating the mitotic nuclei in LCC- HI. The ColonNet method has two phases: initially, the possible mitotic patches in the HI regions are recognized after which these patches are classified into adenocarcinomas (colon), benign (colon), benign (lung), adenocarcinomas (lung), and squamous cell carcinomas, according to the model’s strategies. The author employed and developed deep CNNs while each layer took different textural, morphological, and structural cancer nuclei properties to construct the heteromorphous deep CNNs. The presented ColonNet method was implemented and compared against the advanced CNNs. AlGhamdi et al. [16] developed the BERTL-HIALCCD method to identify the LCC from HI in an effective manner. To perform this, the BERTL-HIALCCD technique encompassed CV and TL models for precise LCC recognition. While utilizing the BERTL-HIALCCD method, an enhanced ShuffleNet method was also used to extract the features. Further, its hyperparameters were fine-tuned using the BER method. Then, the DCRNN method was used to identify the LCC effectively. At last, the COA was used for the selection of the parameters using the DCRNN method. 

Al-Jabbar et al. [17] proposed three advanced techniques, each of which employed two methods for the initial diagnosis of LC25000 dataset histological images. In this study, the histological images were enhanced whereas the outcomes attained from the comparison of the affected regions were also improved. The VGG-19 and GoogLeNet methods of every system formed higher dimensional features; therefore, unnecessary and redundant features were eliminated using the Principal Component Analysis (PCA) technique to get rid of the high dimensionality issue and retain only the necessary features. The initial tactic for analyzing the LC25000 dataset HI by ANN made use of the significant features present in the VGG-19 and GoogleNet methods. While a few systems reduced the whole number of dimensions and united it, the rest of the systems united a higher number of features, eventually resulting in fewer higher dimensions. Kumar et al. [18] used the NLM filter to restrain the impact of noise from the HI. The NLM filter effectually reduced the noise while maintaining the image edges. The attained denoised images were used as input to the presented ML3CNet. Additionally, the quantization method was also utilized to reduce the size of the presented model for storing the data. 

## 3. Proposed Approach

**Figure 1:**
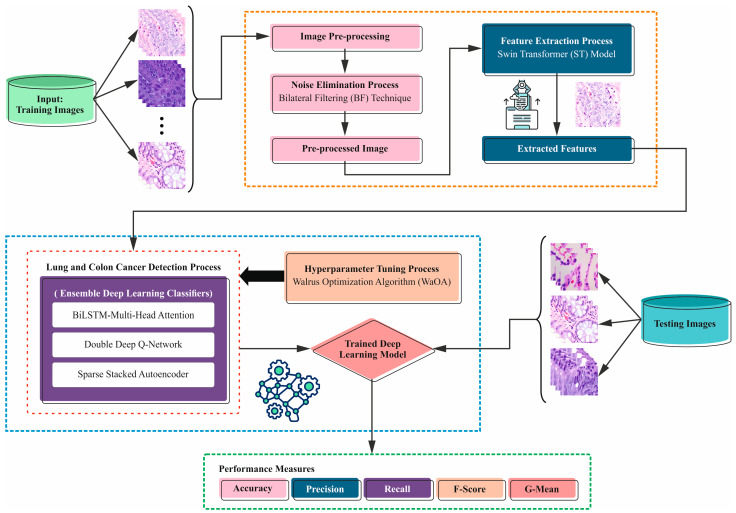
Workflow of the LCCST-EMHI model.

This study proposes a novel LCCST-EMHI technique. The proposed technique uses histopathological images to classify and recognize the LCC. In order to achieve this, the LCCST-EMHI model contains distinct stages as listed herewith: image preprocessing, feature extractor, ensemble learning, and WaOA-based parameter selection. Figure 1 denotes the working flow of the proposed LCCST-EMHI model.

### 3.1. Image Preprocessing

**Figure 2:**
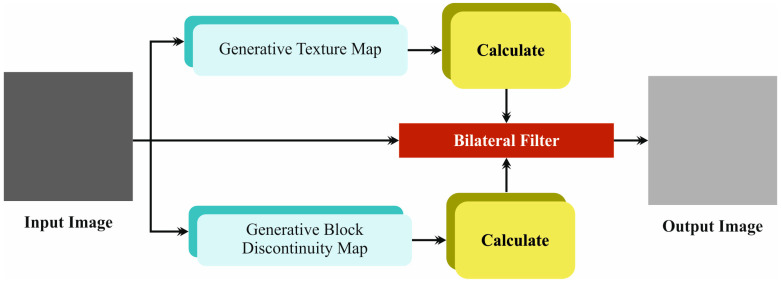
Structure of the BF model.

In the initial phase, the LCCST-EMHI method uses the bilateral filtering (BF) model to get rid of the noise [19]. The BF model is often preferred for image preprocessing tasks as it can conserve the edges while smoothening the noise in parallel. Unlike other mean filters that blur all the pixels in a uniform manner, BF utilizes spatial and intensity data to selectively smoothen the regions of similar intensity while at the same time maintaining sharp boundaries. This edge-preserving quality is a significant feature, useful in applications that demand the retention of detail, for instance, medical imaging or computer vision tasks. Furthermore, the capability of the BF technique to adapt to different levels of noise and detail makes it a versatile and effective candidate for a wide range of images. It also confirms the overall improvement in image quality and highly accurate subsequent processing. Figure 2 portrays the structure of the BF model.

In general, the input images have much noise, and so it becomes essential to remove the noise from these images at the initial stages. In this study, the BF is utilized for noise reduction. Bilateral filtering, the edge-preserving smoothing technique is a non-repeating process. Unlike conventional mean filters that compute the average value of the pixels in a neighbourhood, the BF conserves the edges by considering both spatial distances as well as the intensity differences between the pixels. While the standard mean and the median filters may blur or reduce the edges and noise, the BF thrives to smoothen the image without compromising on the crucial edge data. This characteristic helps in preventing excessive reduction in the edge details and so, the overall structure of the image. The non-linear bilateral filter has been advanced to tackle the problem discussed above. A bilateral filter, utilizing linear Gaussian G as the initial step for smoothening the edges, is given in Equation (1).

(1) $$Gx=(f\times LG_{S})(x)$$

The weight for $f\left(y\right)$ equates to $LG_{S}(x-y)$ and is calculated based on the spatial distance, $\left\|x-y\right||$. The BF improves a weight term wt based on the complete distance denoted by $f(y)-f\left(x\right)$.

These outcomes are briefed in Equation (2).

(2) $$G(x)=\int f(y)\times LG_{S}\left(x-y\right)\times wtdy$$

It is to be understood that when the weight instantly depends on the image values, normalization becomes an essential step to ensure that the overall weights are equivalent to one. The main advantage of BF is that it represents a noise deduction process by image smoothening.

### 3.2. Feature Extractor

**Figure 3:**
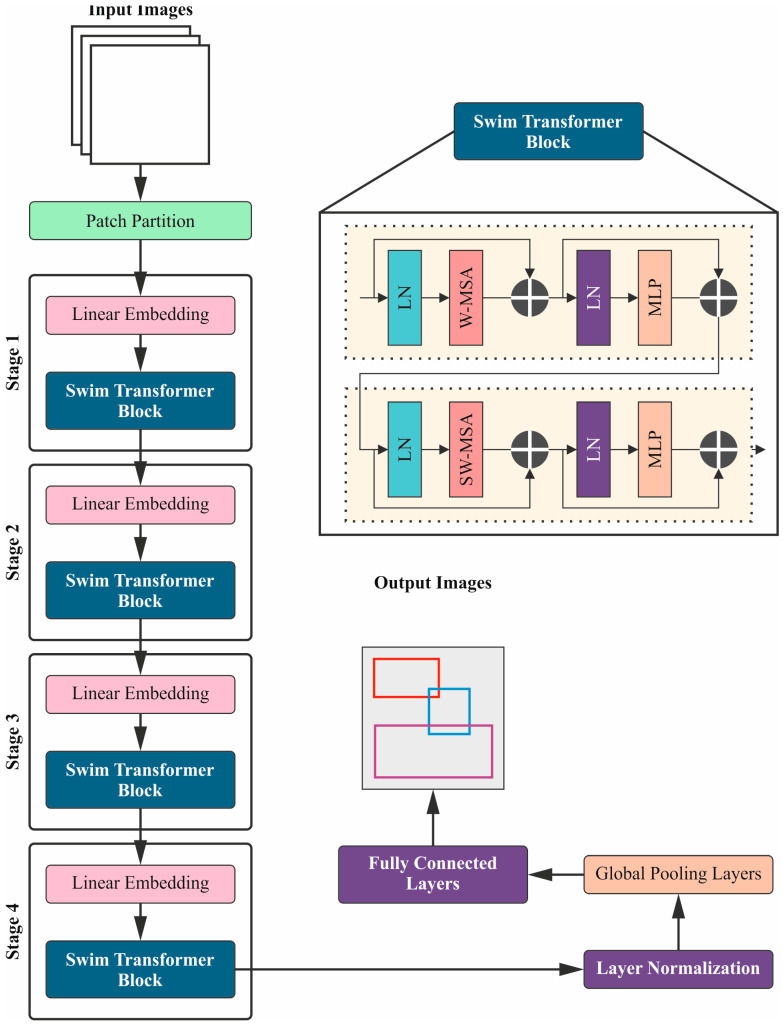
Stages involved in the ST technique.

Next, the LCCST-EMHI technique uses the ST (Swin Transformer) model for the purpose of feature extraction [20]. The ST method is highly effective in the feature extraction process, owing to its hierarchical design and local-to-global processing abilities. Unlike the conventional CNNs, the STs utilize shifted windows to capture both local as well as the global features in an effective manner. This methodology allows the model to scale according to diverse resolutions and capture all the intricate details while maintaining computational efficiency. Moreover, the capability of the ST model in terms of handling various input sizes and its strong performance in capturing long-range dependencies make it a robust choice for feature extraction process in complex tasks. The current section provides an elaborate explanation of the ST method. It employs a hierarchical feature map structure through shifted windows, a technique that maintains linear computational complexity. The system also implements a four-stage ST model. Primarily, the input image is resized before it is fed into the ST model. Within the ST model, the image undergoes processing through various modules, thus resulting in a pool of refined and well-focused output feature maps. An image with an input size measured at 224×224×3 is handled with a standard 2×22D convolution (Conv) layer for extracting crucial features. This convolutional layer extracts the essential features from the image using a 2 × 2 kernel across the overall image. This task helps in capturing the local patterns as well as textures, a significant feature for subsequent feature extraction and analysis. The convolution process efficiently mitigates the spatial dimensions while also conserving the substantial data required for additional processing. Figure 3 demonstrates the stages involved in the ST methodology.

1. Feature Map and Patch Division: After the initial 2D convolution layer, the feature map is divided into non-overlapping patches with size measurements being H×W. Here, H and W illustrate the height and width of each patch, respectively. This sort of dividing into patches is a useful technique for handling image data in a structured manner as it assists in processing and analyzing smaller and localized areas of the image in an effective manner.
2. ST Blocks: The patches are then processed through a series of ST blocks. The ST blocks are constructed to handle both local as well as global features through self-attention mechanisms. Each ST block employs either Shifted Window Multi-Head Self-Attention (SW-MSA) or Window Multi-Head Self-Attention (W-MSA) to capture the long-range dependencies as well as the global features. Here, the SW-MSA integrates the attention within the shifted windows to improve the outcomes in terms of capturing the features, while W-MSA focuses on fixed, non-overlapping windows.
3. Patch Fusion and Linear Layer: After processing the image through the ST blocks, the adjacent sets of 2 × 2 patches are integrated using patch fusion layers. This step incorporates the features from neighbouring patches to provide a highly comprehensive understanding of the image. The incorporated features are then processed using a linear layer, which assists in further transformation and reduction in the dimensions.
4. Down-sampling and Final Dimension: The feature map undergoes down-sampling by a factor of 2 as it mitigates the spatial dimensions. If the original dimension of the feature map is H×W, then the final output of the feature map dimension becomes $\frac{H}{32}\times \frac{W}{32}\times 8C$, where $\frac{H}{32}\times \frac{W}{32}$ reflects the reduced height and width, respectively, after multiple down-sampling operations. Here, 8C corresponds to the number of channels in the final feature map in which C denotes the base number of channels after the convolution and attention processes. In this context, 8C means eight times the base number of channels, which translates into the fact that it provides a rich representation of the features in the final output.

Hence, the feature map is first divided into patches. Then, the patches are processed through ST blocks with attention mechanisms followed by fusing and dimensionality reduction stages, which eventually results in a final feature map with dimensions altered according to the captured crucial data. 

### 3.3. Ensemble Learning

In addition to the above-discussed methods, the LCC is also detected and classified using an ensemble of three classifiers namely, BiLSTM-MHA, DDQN, and SSAE. The sub-sections provide an in-depth insight into all three classifiers. 

#### 3.3.1. BiLSTM-MHA Classifier

RNN is a method employed to handle sequence data [21]. Generally, the LSTM is an advanced version of the RNN method with interior methods comprising forget, output, and input gates. The forget gate manages the present output and input of the preceding Hidden Layer (HL). The input gate defines the criteria for the data to be used as an input into the system and the output gate selects the data from a cell state that desires to be the output. 

Here, $I_{t}$, $f_{gt},\;i_{gt}$, $O_{gt}$, $h_{t}$, $C_{t}$, and ${\tilde{C}}_{t}\;$correspond to input data, forget gate, input gate, output gate, present HL, present output, and temporary state of a cell at tth time, respectively. In LSTM networks, t denotes a specific time step or position in a sequence. During every $t^{th}$ time step, the network processes the input data, updates its cell state, and yields an output based on the current and previous states. This allows the LSTM network to capture the temporal dependencies in sequential data for which the formulations are given below.

(3) $$f_{gt}=\sigma \left(W_{fh}\cdot h_{t-1}+W_{fI}\cdot I_{t}+b_{f}\right)$$

(4) $$i_{gt}=\sigma \left(W_{ih}\cdot h_{t-1}+W_{iI}\cdot I_{t}+b_{i}\right)$$

(5) $$O_{gt}=\sigma \left(W_{oh}\cdot h_{t-1}+W_{oI}\cdot I_{t}+b_{O}\right)$$

(6) $$h_{t}=O_{gt}*tanh\left(C_{t}\right)$$

(7) $${\tilde{C}}_{t}=tanh\left(W_{Ch}\cdot h_{t-1}+W_{CI}\cdot I_{t}+b_{C}\right)$$

(8) $$C_{t}=f_{gt}*C_{t-1}+i_{gt}*{\tilde{C}}_{t}$$

Here, W represents the weight matrix, b denotes a bias parameter and σ(z) refers to a sigmoid activation function. The formulations for σ(z) and tanh(x) are given in Equations (9) and (10).

(9) $$\sigma (z)=\frac{1}{1-e^{-z}}$$

(10) $$tanh(x)=\frac{e^{x}-e^{-x}}{e^{x}+e^{-x}}$$

The Bi-LSTM network can utilize the data from previous or future moments, thus efficiently using the dependence between future and historical time-step data.

The Bi-LSTM backward layer depends on the future time-step data. The forward layer permits the method to incorporate both past as well as imminent time-step data for predicting the future. Therefore, the Bi-LSTM network improves the representation of feature ability and also obtains enriched sequence aspects.

**Figure 4:**
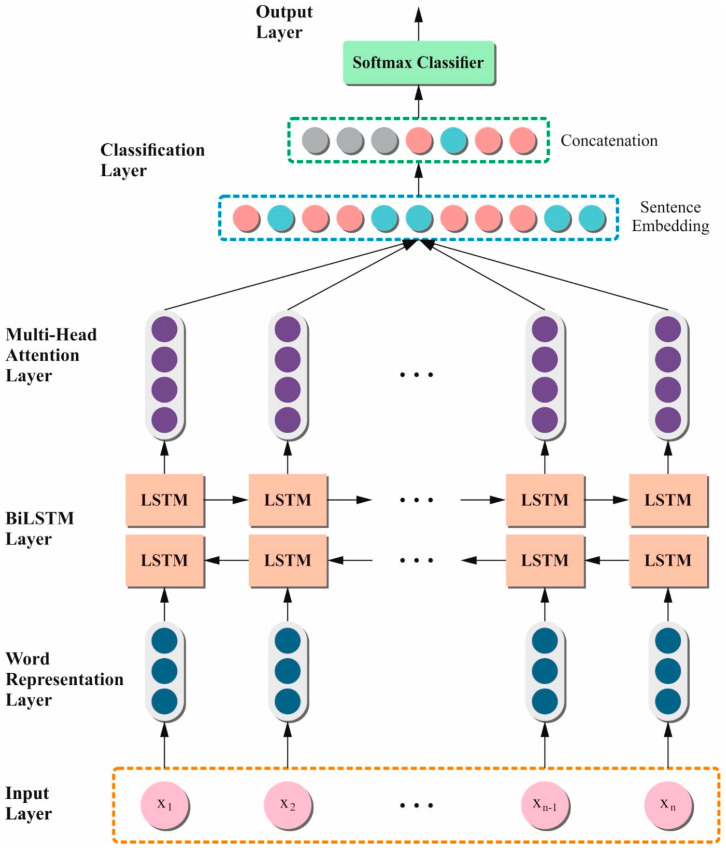
Structure of the BiLSTM-MHA method.

The multi-head attention method acquires dissimilar weight parameters by loading a self-attention mechanism at multiple folds. For a similar input X, numerous arrays of dissimilar parameter matrices are employed to achieve dot-product processes by input so as to attain dissimilar attention matrices such as K, Q, and V. Figure 4 denotes the structure of the BiLSTM-MHA technique. Next, every attention head output is integrated and directly changed. This procedure is expressed in the equation given below.

(11) $$MHA\left(Q,K,V\right)=Concat\left(Head_{1}\ldots Head_{n}\right)7V^{0}$$

Meanwhile, $Head_{i}$ denotes a single self-attention mechanism.

#### 3.3.2. DDQN Classifier

DQN is an off-policy and model-free learning technique that seeks optimum policies in terms of agent contact [22]. A set of rewards is often referred to as R, a set of states is denoted by S, and a set of actions is signified as A at every time step and obtains a state s∈S and performs an action a∈A, which results in the subsequent state $\left(s^{\prime }\right)$ and immediate reward (r). This successive procedure develops a route by signifying the states, rewards, and actions that are maintained with regained experience. The value of the target state-action depends on the subsequent state-action, which is set with an immediate reward $\left(s^{\prime },\;a\;Q_{t}(s,\;a)=r+\gamma maxQ(s^{\prime },\;a^{\prime }\right)$. On the contrary, γ denotes the discount factor that considers future rewards. The DQN model utilizes both the experiences’ replay and target network, where the target network $\left(\hat{Q}\right)$ with parameter $\theta^{-}$ is similar to the online network. Still, its limitations are reproduced with definite ranges from the online network and are retained and fixed during every other step. Equation (12) shows the calculation for DQN.

(12) $$Q_{target}^{DQN}\equiv r+\gamma {max}_{a^{\prime }}\hat{Q}\left(s^{\prime },a^{\prime };\theta^{-}\right)$$

During the training process, instead of upgrading the Q-network with current practices that might have time-based correlations, a randomly generated set of experiences is tested from the experiences gained earlier. Conversely, the preceding calculation is also carried out based on an underestimation of the Q-value. The DDQN tackles the restriction using a dissimilar network so as to evaluate the preferred action. This scenario dissociates the range from the assessment to performance enhancement.

(13) $$Q_{target}^{DDQN}\equiv r+\gamma \hat{Q}\left(s^{\prime },\;{max}_{a^{\prime }}Q\left(s^{\prime },\;a^{\prime };\theta \right);\theta^{-}\right)$$

Here, $\theta^{-}$ and $\hat{Q}\;$represent the weight and target network correspondingly.

**Figure 5:**
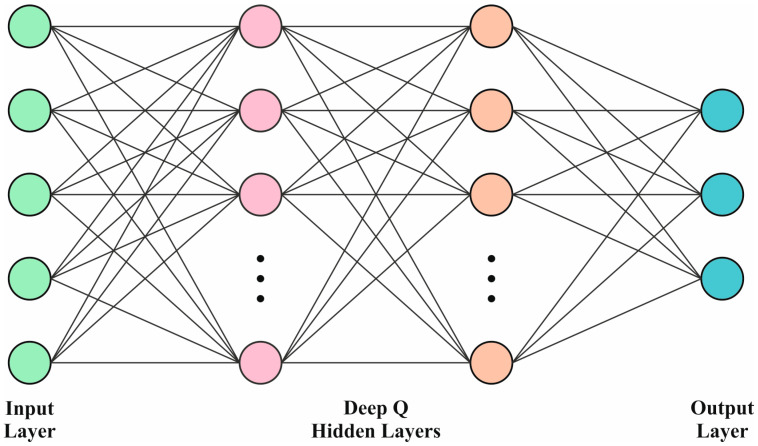
Architecture of the DDQN model.

The performances of the DDQN and DQN techniques are decided by the complexity of the hyperparameters. So, the hyperparameters should be optimized to achieve high performance from the DDQN model. This can be carried out using CNN by reflecting a randomly formed grid range. Batch size, filter size, updated frequency, rate of learning, and the quantity of experiences are vital to acquire, altogether reflecting the importance of optimizing the hyperparameters. Ray Tune Python library was employed in this study to mechanically hunt for the optimum hyperparameters utilizing the hyperopic technique for ten numerous mixtures to boost the mean reward for 1000 episodes. The complete training procedure had 2000 episodes among which only 50 percent were reflected, owing to a comprehensive computation time. Figure 5 illustrates the architecture of the DDQN model.

To project the noise ablation structure as a separate entity, the current study utilized DDQN with CNN with the optimized hyperparameters. For contrast intents, the FCN was studied with dimensions equal to the CNN filter size i.e., hidden layer (HL) with 64, 32, and 128 neurons. Both output and input layers were maintained in parallel for both cases and were equivalent to the action and state space sizes. Further, the study also used linear activations and ReLU for the HL, CNN output layer, and FCN. The convolutional layer utilized the kernel with 2 × 2 dimensions containing 2, 2, and 1 steps correspondingly. For the reflection and transmission issues, both DDQN and DQN methods share parallel network hyperparameters and architectures with performance variation primarily based on the target analysis of the Q-value.

#### 3.3.3. SSAE Classifier

SSAE classifier is a hierarchical DNN that contains deep AE. The primary AE structure contains output, input, and hidden layers [23]. Here, the encoding process is responsible for the extraction of hidden features from the input data, though the decoding process recreates the input data by utilizing the HL features. By corresponding to the input data, the AE maintains the essential features. Then, the AE input layer obtains the data whereas the transitional layers create hidden codes from the input data. When the input data undergo downsampling by PCA, hidden codes are generated whose dimensions depend on the nodes in HL. The last layer decodes these hidden codes to restructure the first input. The AEs alter the input data into hidden codes and utilize them for restructuring purposes. The usual representation for the AE input is $x=[x_{1},x_{2},\;\ldots ,x_{n}].$ T in RD while $D_{x}$ represents the input size. During the encoding process, the input x is decoded to the HL feature vector h and it is comprised of $D_{h}$ neurons in the hidden layer. This alteration method uses an activation function, f. The encoding layer encrypts the network input, whereas the decoding layer decrypts them. The main goal of the AE classifier is to calculate the reconstructed code h to recover it with high precision and it is mathematically expressed in Equation (14).

(14) $$f_{dec}\left(f_{enc}\left(x\right)\right)=f_{dec}\left(h\right)=X\approx X$$

**Figure 6:**
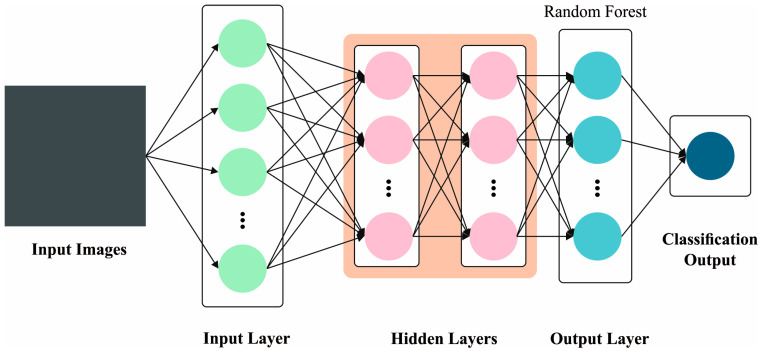
Architecture of the SSAE approach.

Meanwhile, $f_{enc}$ signifies the encoding layer function, and $f_{dec}$ denotes a decoding functional layer. The number of neurons in the encoding layer is usually low compared to the input dimension. Therefore, the system is stimulated to reduce the input dimension in this layer so as to avoid redundancy. The usual backpropagation (BP) method with weight initialization at random is compatible by training a distinct AE since it is a shallow neural network. Figure 6 portrays the structure of the SSAE model.

### 3.4. Parameter Selection

**Figure 7:**
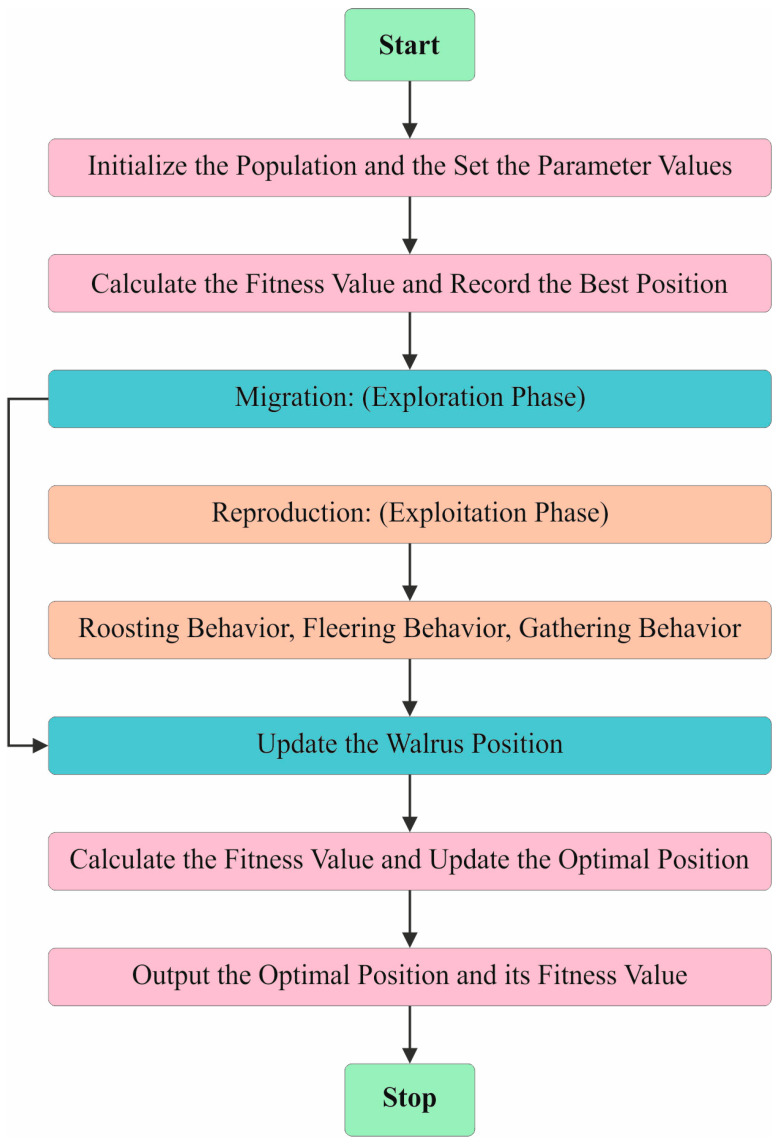
Steps involved in WaOA technique.

Finally, the hyperparameter selection of all three DL models is executed using the walrus optimization algorithm (WaOA). The WaOA model is a novel method inspired by the natural behaviour of the walrus that presents various merits for hyperparameter tuning [24]. It yields excellent performance in complex search space exploration tasks by employing diverse population dynamics and adaptive mechanisms. The balance between the exploration and exploitation phases in this WaOA technique not only assists in efficiently navigating the hyperparameter landscape but also avoiding the local optima issue. Further, it also enhances the convergence of the model towards the optimal solution. Compared to other methods, the WaOA method can produce highly robust and accurate tuning by effectually handling the high-dimensional spaces and diverse hyperparameter configurations. It has the capability to adapt to diverse problem domains and incur relatively low computational costs, which in turn improves its merit in hyperparameter optimization tasks. It simulates their social ability structures to attain effective optimization. Walruses generally have a vital intelligence of touch. Also, the technique respects the social hierarchies and structures followed by the adults, females, and juvenile walruses in their groups. These fundamental characteristics direct the WaOAs search approach to create a fine balance between the exploitation and exploration phases. Figure 7 shows the steps involved in the WaOA method. The upcoming segment details the foremost models of the WaOA method.

The WaOA optimization procedure starts by producing an initial population of the candidate solutions that are randomly generated to drop in the predefined lower and upper restraints for the optimization issue variables. This dissimilar initial point certifies that there is sufficient exploration space for attaining a latent optimum solution. During the WaOA iterations, these agents travel according to environmental and social signals and always concentrate on the finest potential solution.

By using danger and safety signals, the WaOA technique simulates the responsive behaviour of the walruses according to their atmosphere. These kinds of signals impact the behaviour of each agent and direct the population near regions, where the finest solution is probable to originate. The danger signal imitates the danger related to an agent’s present location, which is determined using Equation (15). In case of improved optimization, this risk signal progressively fails and encourages the agents to travel near the possible solutions. Equation (19) calculates the protection signal, which reveals the appeal of an agent’s present position and it also acquires the robustness during each iteration, thus promising excellent exploitation outcomes. This technique equalizes these densities in an efficient manner so as to navigate the search space.

(15) $$Danger_{-}signa1=A\times R\;$$

(16) A=2×α

(17) $$\alpha =1-\frac{t}{T}$$

(18) $$R=2\times r_{1}-1$$

(19) $$Safety\_signal=r_{2\;}$$

There are dual risk factors signified by R and A. The parameter α reduces from 1 to 0 in the optimization procedure. The safety signal is denoted by $r_{2}$, while the remaining dual stochastic variables, $r_{1}$ and $r_{2}$, fall between 0 and 1. The variable t specifies the present iteration step while T signifies the highest predefined iteration count.

During the migration phase, the walrus agents try to travel to novel regions of the searching space. This is accomplished by reorganizing the location of every agent utilizing a randomly produced number $r_{3}$ and a migrate step β. The below-mentioned calculation signifies how the location of the walrus is upgraded in this stage.

(20) $$X_{ij}^{t+1}=X_{ij}^{t}+Migration_{-}step$$

(21) $$Migration_{-}step=(X_{m}^{t}-X_{n}^{t})\times \beta \times r_{3}^{2}$$

(22) $$\beta =1-\frac{1}{1+e^{\frac{-10(t-0.5T)}{T}}}$$

Here, the adapted position in the ith iteration beside the jth dimension is signified as $X_{ij}^{t+1}$. Dual nominated locations are specified at random by $X_{m}^{t}$ and $X_{n}^{t}$, respectively.

The reproduction phase is the phase of population divergence and in this phase, three clusters of walruses i.e., females, males, and juveniles, show dissimilar behaviours. Male walruses perform as spies and discover novel regions within the search space. Their locations are adapted as desirable ones. Meanwhile, female walruses mainly concentrate on refining the latent thoughts of over-exploitation. Young walruses carry huge differences and their locations are attuned towards guiding their relations with both parents. Both exploration and exploitation features are conjoined in this behaviour, whereas changeability is inserted to allow more exploration.

The WaOA method’s capability to discover the optimum solutions is improved by striking a balance between the discovery of novel areas and the application of promising solutions using numerous reproduction models. Equations (23)–(25) show the calculation for this phase.

(23) $$female_{ij}^{t+1}=female_{ij}^{t}+\alpha \times (male_{ij}^{t}-female_{ij}^{t})+(1-\alpha )\times (X_{best}^{t}-female_{ij}^{r})$$

(24) $$Juvenile_{ij}^{t+1}=(O-Juvenile_{ij}^{t})\times P\;$$

(25) $$O=X_{best}^{t}+Juvenile_{ij}^{t}\times LF\;$$

Assume P signifies the risk factor for young walruses and O denotes the protection target for their location.

The WaOA method develops a Fitness Function (FF) to enhance the classification performance. It identifies a positive number to signify the greater performance of the candidate solution. In this research, reduced classifier rate of error is examined as FF and is shown in Equation (25).

(26) $$fitness\left(x_{i}\right)=ClassifierErrorRate\left(x_{i}\right)\;=\frac{no.\;of\;misclassified\;samples}{Total\;no.\;of\;samples}\times 100$$

## 4. Performance Validation

**Figure 8:**
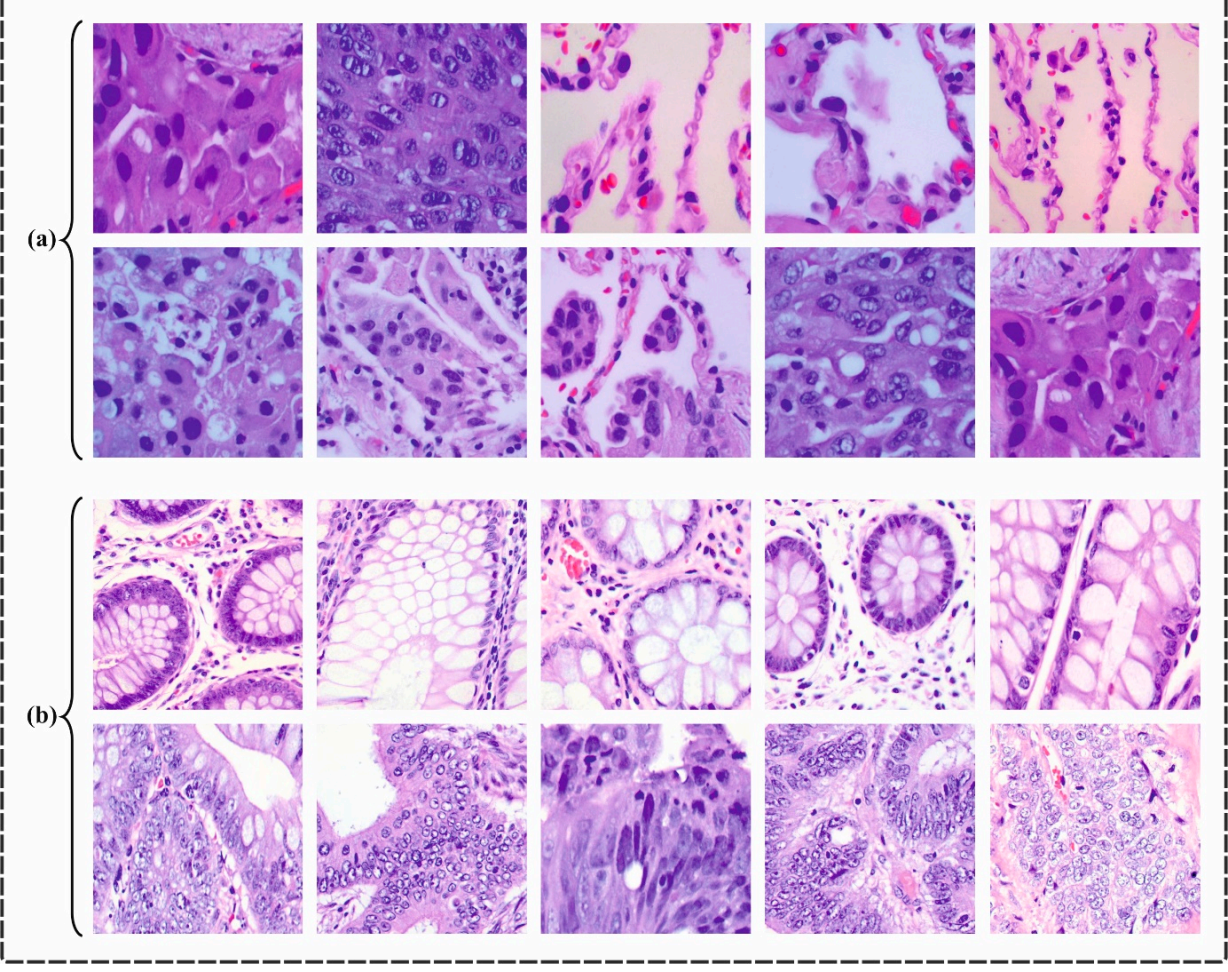
Sample images of (**a**) lung cancer and (**b**) colon cancer.

In this section, the proposed LCCST-EMHI approach was validated using an established dataset through simulation [25]. The dataset encompasses 25,000 histopathological images and each image had the following dimensions: 768 × 768 pixels and stored in JPEG format. Initially derived from a HIPAA-compliant sample of 750 images of lung and colon tissues, around 250 images each from benign and malignant lung adenocarcinomas, lung squamous cell carcinomas, and benign colon tissues, and 500 images of colon adenocarcinomas—the dataset was augmented to 25,000 images by utilizing the Augmentor package. It is then organized into five classes, with 5000 images per class: lung benign tissue, lung adenocarcinoma, lung squamous cell carcinoma, colon adenocarcinoma, and colon benign tissue. Figure 8 presents some of the sample images for lung and colon cancer. The proposed technique was simulated using Python 3.6.5 tool configured on a PC with specifications such as i5-8600k, 250 GB SSD, GeForce 1050Ti 4 GB, 16 GB RAM, and 1 TB HDD. The parameter settings considered for the study are learning rate: 0.01, activation: ReLU, epoch count: 50, dropout: 0.5, and batch size: 5. Table 1 provides a brief description of the dataset. 

**Figure 9:**
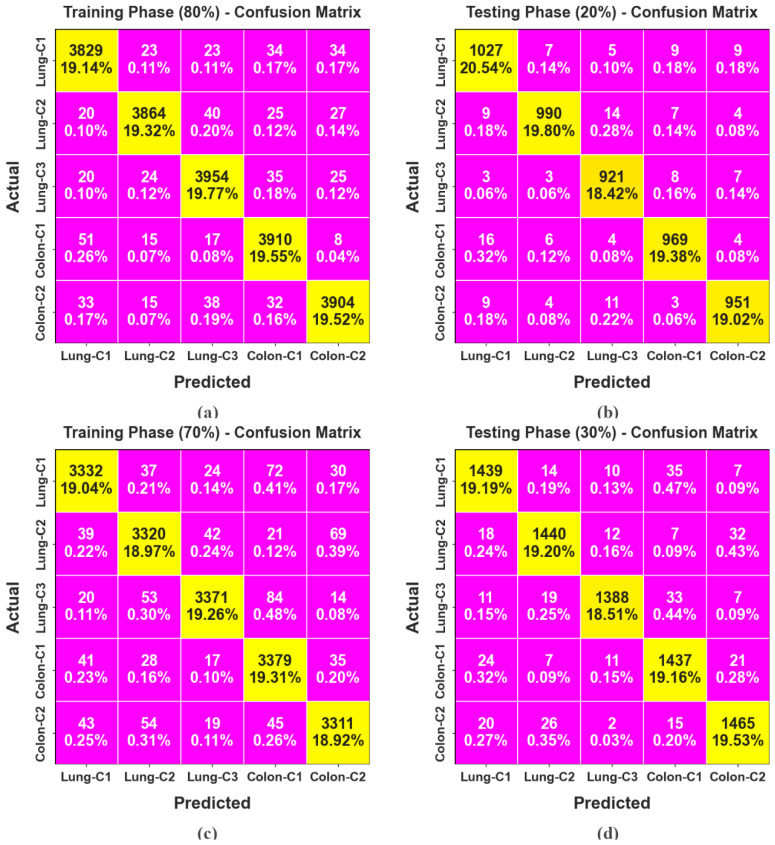
Confusion matrices of (**a**,**c**) 80%TRAP and 70%TRAP and (**b**,**d**) 20%TESP and 30%TESP.

Figure 9 portrays the confusion matrices generated by the LCCST-EMHI approach for 80:20 and 70:30 of TRAP/TESP datasets. The results specify that the proposed LCCST-EMHI approach accurately detected and identified all five classes.

**Figure 10:**
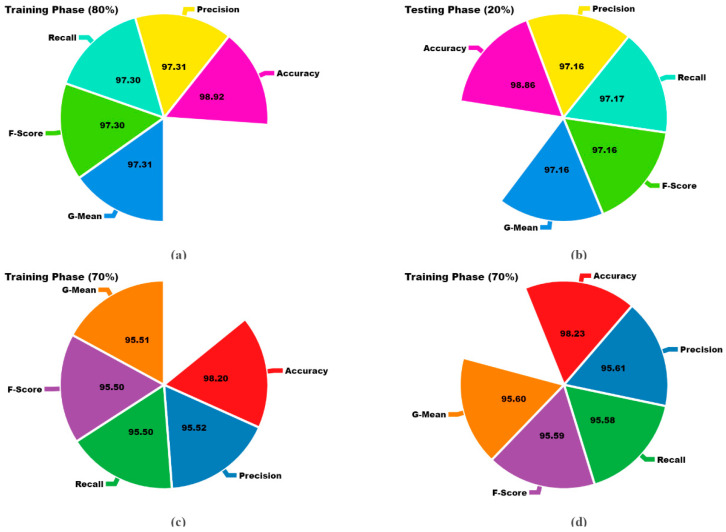
Average values attained by the LCCST-EMHI method for 80:20 and 70:30 of TRAP/TESP datasets (**a**) 80%TRAP, (**b**) 20%TESP, (**c**) 70%TRAP, and (**d**) 30%TESP.

Table 2 and Figure 10 show the cancer detection results attained by the LCCST-EMHI approach under 80:20 and 70:30 of the TRAP/TESP dataset. The table values imply that the LCCST-EMHI technique appropriately identified all five classes. With 80%TRAP, the LCCST-EMHI approach achieved the average $accu_{y}$, $prec_{n}$, $reca_{l}$, $F_{score}$, and $G_{Mean}$ values such as 98.92%, 97.31%, 97.30%, 97.30%, and 97.31%, respectively. With 20%TESP, the LCCST-EMHI method produced the average $accu_{y}$, $prec_{n}$, $reca_{l}$, $F_{score}$, and $G_{Mean}$ values such as 98.86%, 97.16%, 97.16%, 97.16%, and 97.16%, respectively. Additionally, with 70%TRAP, the LCCST-EMHI approach yielded the average $accu_{y}$, $prec_{n}$, $reca_{l}$, $F_{score}$ and $G_{Mean}$ values such as 98.20%, 95.52%, 95.50%, 95.50%, and 95.51%, correspondingly. Moreover, with 30%TESP, the LCCST-EMHI approach accomplished the average $accu_{y}$, $prec_{n}$, $reca_{l}$, $F_{score}$, and $G_{Mean}$ values such as 98.23%, 95.61%, 95.58%, 95.59%, and 95.60%, respectively.

**Figure 11:**
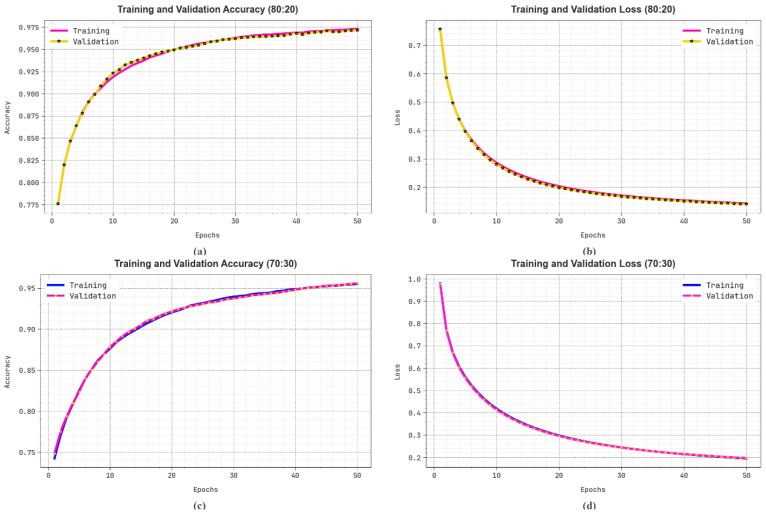
(**a**,**c**) Accuracy curve on 80%:20% and 70%:30% and (**b**,**d**) loss curve on 80%:20% and 70%:30%.

Figure 11 illustrates the classification performance of the LCCST-EMHI model on 80%:20% and 70%:30% datasets. Figure 11a–c show the precision outcomes of the LCCST-EMHI model. The outcome confirms that the LCCST-EMHI model achieved high values over the increasing number of epochs. Additionally, the increased validation over the training dataset confirms that the LCCST-EMHI method has the ability to learn proficiently on the test dataset. On the other hand, Figure 11b–d demonstrates the loss investigation of the LCCST-EMHI technique. The results denote that the LCCST-EMHI model scored closer training and validation loss values and its learning proficiency about the dataset can be understood. 

**Figure 12:**
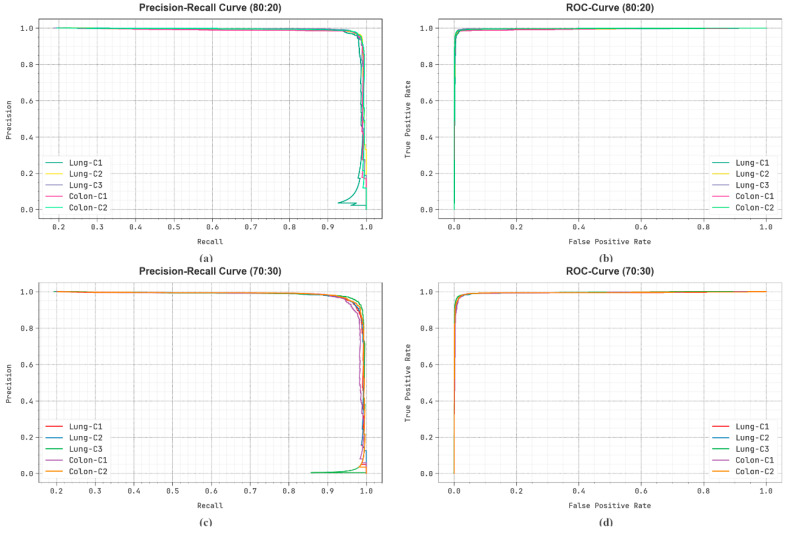
(**a**,**c**) PR curve on 80%:20% and 70%:30% and (**b**,**d**) ROC curve on 80%:20% and 70%:30%.

Figure 12 shows the classification outcomes attained by the LCCST-EMHI methodology on 80%:20% and 70%:30% datasets. Figure 12a–c illustrates the PR outcomes attained by the LCCST-EMHI process. The results infer that the LCCST-EMHI method achieved proficient PR results. Moreover, the LCCST-EMHI methodology has been established to attain high PR outcomes on all the class labels. Lastly, Figure 12b–d shows the ROC curve plotted for the LCCST-EMHI approach. The figure infers that the LCCST-EMHI approach resulted in enhanced ROC values. Also, the LCCST-EMHI model can prolong higher ROC values in every class.

**Figure 13:**
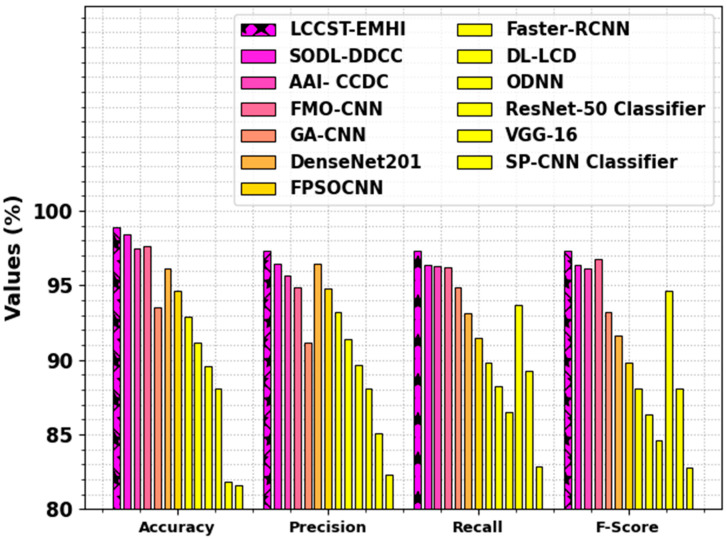
Comparative analysis outcomes of the LCCST-EMHI technique and other existing models.

Table 3 and Figure 13 showcase the comparison outcomes of the LCCST-EMHI process with that of the existing models [26,27,28,29,30]. The outcomes highlight that the SP-CNN, VGG-16, ResNet-50, and GA-CNN models reported worst performance. Meanwhile, the FMO-CNN, AAI- CCDC, and the SODL-DDCC techniques attained closer results. However, the LCCST-EMHI approach reported superior performance with the maximum $prec_{n}$, $reca_{l},$ $accu_{y},$ and $F_{score}$ values such as 97.31%, 97.30%, 98.92%, and 97.30%, respectively.

**Figure 14:**
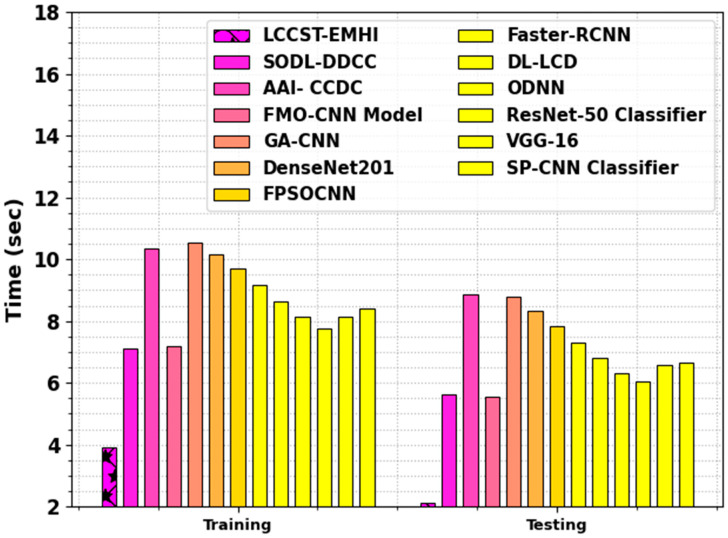
Training and testing time comparative analysis between the LCCST-EMHI model and other recent models.

In Table 4 and Figure 14, the comparison outcomes of the LCCST-EMHI approach with that of the other models are shown with regard to training and testing time. The outcomes suggest that the LCCST-EMHI model achieved optimal performance. In terms of training time, the LCCST-EMHI technique consumed the least training time of 3.92 s, whereas the SODL-DDCC, AAI- CCDC, FMO-CNN, GA-CNN, ResNet-50, VGG-16, and SP-CNN approaches consumed lengthy training time such as 7.11 s, 10.35 s, 7.20 s, 10.53 s, 10.17 s, 9.70 s, 9.16 s, 8.62 s, 8.16 s, 7.75 s, 8.15 s, and 8.41 s, respectively. In terms of testing time, the LCCST-EMHI process took the least testing time of 2.14 s, whereas the SODL-DDCC, AAI- CCDC, FMO-CNN, GA-CNN, ResNet-50, VGG-16, and SP-CNN methods required lengthy testing time such as 5.61 s, 8.85 s, 5.57 s, 8.80 s, 8.32 s, 7.83 s, 7.31 s, 6.81 s, 6.30 s, 6.04 s, 6.57 s, and 6.67 s, correspondingly.

## 5. Conclusions

In this research, a new LCCST-EMHI technique has been proposed. The proposed LCCST-EMHI method makes an effort to use the DL model for the detection and classification of LCC using the HI. The LCCST-EMHI model contains distinct stages such as image preprocessing, feature extraction, ensemble learning, and WaOA-based parameter selection to accomplish the objective. Primarily, the LCCST-EMHI method utilized the BF technique for noise elimination. Then, the ST model was deployed for the purpose of feature extraction. An ensemble DL classifier containing three techniques namely, BiLSTM-MHA, DDQN, and SSAE, was also used for LCC recognition and identification process. Eventually, the hyperparameter selection of the three DL models was implemented utilizing the WaOA method. In order to illustrate the promising performance of the LCCST-EMHI technique, a wide range of simulation analyses was conducted on the benchmark database. The experimentation results confirm the promising performance of the LCCST-EMHI approach in comparison with the rest of the methods. 

## Figures and Tables

**Table 1:** Details of datasets.

Class Names	Labels	No. of Images
“Lung benign tissue”	Lung-C1	5000
“Lung adenocarcinoma”	Lung-C2	5000
“Lung squamous cell carcinoma”	Lung-C3	5000
“Colon adenocarcinoma”	Colon-C1	5000
“Colon benign tissue”	Colon-C2	5000
Total no. of Images		25,000

**Table 2:** Cancer detection outcomes of the LCCST-EMHI approach for 80:20 and 70:30 of the TRAP/TESP datasets.

Class Labels	$Accu_{y}$	$Prec_{n}$	$Reca_{l}$	$F_{score}$	$G_{Mean}$
TRAP (80%)					
Lung-C1	98.81	96.86	97.11	96.99	96.99
Lung-C2	99.06	98.05	97.18	97.61	97.61
Lung-C3	98.89	97.10	97.44	97.27	97.27
Colon-C1	98.91	96.88	97.73	97.30	97.30
Colon-C2	98.94	97.65	97.07	97.36	97.36
Average	98.92	97.31	97.30	97.30	97.31
TESP (20%)					
Lung-C1	98.66	96.52	97.16	96.84	96.84
Lung-C2	98.92	98.02	96.68	97.35	97.35
Lung-C3	98.90	96.44	97.77	97.10	97.10
Colon-C1	98.86	97.29	97.00	97.14	97.14
Colon-C2	98.98	97.54	97.24	97.39	97.39
Average	98.86	97.16	97.17	97.16	97.16
TRAP (70%)					
Lung-C1	98.25	95.88	95.34	95.61	95.61
Lung-C2	98.04	95.07	95.10	95.09	95.09
Lung-C3	98.44	97.06	95.17	96.11	96.11
Colon-C1	98.04	93.84	96.54	95.17	95.18
Colon-C2	98.23	95.72	95.36	95.54	95.54
Average	98.20	95.52	95.50	95.50	95.51
TESP (30%)					
Lung-C1	98.15	95.17	95.61	95.39	95.39
Lung-C2	98.20	95.62	95.43	95.52	95.52
Lung-C3	98.60	97.54	95.20	96.36	96.36
Colon-C1	97.96	94.11	95.80	94.95	94.95
Colon-C2	98.27	95.63	95.88	95.75	95.75
Average	98.23	95.61	95.58	95.59	95.60

**Table 3:** Comparative analysis outcomes of the LCCST-EMHI approach and other existing models [26,27,28,29,30].

Methods	$Accu_{y}$	$Prec_{n}$	$Reca_{l}$	$F_{score}$
LCCST-EMHI	98.92	97.31	97.30	97.30
SODL-DDCC	98.45	96.44	96.38	96.37
AAI- CCDC	97.48	95.70	96.32	96.17
FMO-CNN	97.65	94.87	96.19	96.76
GA-CNN	93.56	91.15	94.89	93.21
DenseNet201	96.16	96.49	93.16	91.64
FPSOCNN	94.66	94.82	91.49	89.85
Faster-RCNN	92.90	93.19	89.78	88.10
DL-LCD	91.13	91.42	88.26	86.37
ODNN	89.61	89.69	86.49	84.64
ResNet-50 Classifier	88.05	88.08	93.67	94.63
VGG-16	81.82	85.11	89.26	88.10
SP-CNN Classifier	81.61	82.31	82.90	82.75

**Table 4:** Training and testing time comparative analysis between the LCCST-EMHI model and other recent models.

Time (s)		
Methods	Training	Testing
LCCST-EMHI	3.92	2.14
SODL-DDCC	7.11	5.61
AAI- CCDC	10.35	8.85
FMO-CNN Model	7.20	5.57
GA-CNN	10.53	8.80
DenseNet201	10.17	8.32
FPSOCNN	9.70	7.83
Faster-RCNN	9.16	7.31
DL-LCD	8.62	6.81
ODNN	8.16	6.30
ResNet-50 Classifier	7.75	6.04
VGG-16	8.15	6.57
SP-CNN Classifier	8.41	6.67

## Data Availability

The data supporting this study’s findings are openly available in the Kaggle repository at https://www.kaggle.com/datasets/andrewmvd/lung-and-colon-cancer-histopathological-images, reference number [25].
